# TIM-3 and γδ T cells: new players in breast cancer dissemination

**DOI:** 10.1038/s44318-025-00550-w

**Published:** 2025-08-26

**Authors:** Lorenzo Galluzzi, Claudia Galassi, David L Wiest

**Affiliations:** 1 Cancer Signaling and Microenvironment Program, Fox Chase Cancer Center, Philadelphia, PA USA; 2 Department of Pharmacology, Weill Cornell Medical College, Weill Cornell Medical College, New York, NY USA; 3 Nuclear Dynamics and Cancer Program, Fox Chase Cancer Center, Philadelphia, PA USA

**Keywords:** Cancer, Immunology

## Abstract

Recent data report a role for IL-17-secreting γδ T cells in facilitating the establishment of TIM-3^+^ TNBC metastases via local immunosuppression.

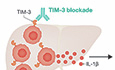

## Teaser

γδ T cells, a heterogeneous lymphocyte subset operating at the interface between innate and adaptive immunity, mediate ambivalent and context-dependent effects in oncological settings. Recent work by Rozalén et al, (2025) demonstrates that IL-17-secreting γδ T cells favor the outgrowth of TIM-3^+^ triple-negative breast cancer metastases by establishing local immunosuppression.

γδ T cells are a population of CD3^+^CD4^-^CD8^-^ lymphocytes that, unlike their αβ lineage CD4^+^CD8^-^ or CD4^-^CD8^+^ counterparts, express a T-cell receptor (TCR) complex that recognizes intact molecular species in an MHC-independent manner, and operate at the functional interface between innate and adaptive immunity (Mensurado et al, 2023). Besides being fundamental for the maintenance of specific epithelial barriers, notably the intestinal epithelium (Ribot et al, 2021), γδ T cells can be found (although generally with limited abundance) in the immunological infiltrate of most (if not all) solid tumors (Gentles et al, 2015). That said, the pathophysiological consequences of γδ T-cell infiltration exhibit a considerable degree of context dependency, as they either attenuate or promote tumor progression depending on effector functions (Mensurado et al, 2023). Indeed, γδ T cells can differentiate toward a spectrum of functional phenotypes ranging from γδT1 effectors, which express CD27, are marked by the lineage-defining transcription factors eomesodermin (EOMES) and T-box transcription factor 21 (TBX21, best known as T-bet), and secrete the anticancer immune effector interferon gamma (IFNG), to γδT17 effectors, which do not express CD27, are marked by the lineage-defining transcription factor RAR related orphan receptor C (RARC, best known as RORγt), and produce the immunomodulatory cytokine interleukin 17A (IL17A, best known as IL-17) (Ribot et al, 2021). Thus, while CD27^+^IFNG^+^ γδ T cells have been associated with active anticancer immune responses in a variety of preclinical and clinical oncological settings, including patients with MHC-negative colorectal cancer treated with immune checkpoint inhibitors (de Vries et al, 2023), CD27^-^IL-17^+^ γδ T cells have consistently been linked with accelerated tumor progression and resistance to therapy, especially (but not exclusively) in breast cancer (Coffelt et al, 2015; Petroni et al, 2025). A recent study by Rozalén and collaborators (2025) extends these latter observations by demonstrating that triple-negative breast cancer (TNBC) cells that express the co-inhibitory receptor hepatitis A virus cellular receptor 2 (HAVCR2, best known as TIM-3) are superior at establishing metastatic lesions compared to their TIM-3^-^ counterparts, at least in part through the interleukin 1 beta (IL1B, best known as IL-1β)-dependent recruitment of IL-17^+^ γδ T cells, which contribute to the establishment of local immunosuppression and overt immunoevasion (Galassi et al, 2024; Rozalen et al, 2025).

First, Rozalen et al (2025) set out to investigate the ability of breast cancer cells to evade immunity during metastatic dissemination by delivering GFP-expressing mouse EpRas breast cancer cells (which have been developed in vitro and hence have not been subjected to immunological selection a priori) intracardially to immunocompetent, syngeneic BALB/c mice vs highly immunodeficient NSG mice. Besides the fact that brain, lung and liver metastatic dissemination was considerably reduced in immunocompetent vs immunodeficient hosts, EpRas breast cancer cells recovered from metastatic disease sites in BALB/c mice exhibited a different transcriptional profile than their counterparts from NSG mice, including increased levels of *Havcr2*. While TIM-3 is mostly (but not exclusively) expressed by immune cells, *Havcr2* mRNA levels were confirmed to be increased in a fraction of human breast cancer cells. Moreover, different mouse breast cancer cells lines were found to be 10–40% positive for TIM-3 expression by flow cytometry. Finally, TIM-3 levels were also elevated in hepatic metastases established by the intracardiac injection of mouse 4T07 TNBC cells in immunocompetent vs immunodeficient hosts, globally pointing to a role for TIM-3 expression by malignant cells in the establishment of metastatic lesions in the context of active immunosurveillance (Rozalen et al, 2025). In line with this possibility, the selective depletion of TIM-3 from 4T07 TNBC cells (as well as in other mouse models of TNBC) reduced metastatic dissemination to the brain, lung and liver upon intracardiac injection, *de facto* extending the survival of BALB/c (but not NSG) mice receiving such as a challenge. Moreover, the knockdown of TIM-3 limited spontaneous metastatic dissemination following the orthotopic inoculation of mouse TNBC 4T1 cells to the mammary fat pads, with no alteration in the growth of primary tumors. Finally, TIM-3 levels were found to be increased in metastatic vs matched malignant lesions from 75 patients with breast cancer (Rozalen et al, 2025).

To elucidate the molecular mechanisms underlying these observations, Rozalen et al (2025) analyzed the transcriptome of wild-type vs. TIM-3-depleted 4T07 cells isolated from lung and liver metastases two weeks after intracardiac delivery, detecting an enrichment in signatures linked to stemness-related processes, such as the epithelial-to-mesenchymal transition (EMT) and WNT/β-catenin signaling. In line with this notion, TIM-3-depleted 4T07 metastases exhibited reduced expression of EMT markers, including vimentin (VIM) and cadherin 2 (CDH2). Functional assays confirmed that TIM-3 levels (as experimentally reduced or augmented) positively correlated with stemness features in 4T07 cells, including sphere-forming capacity and in vivo tumor formation, at least in some assays in a catenin beta 1 (CTNNB1)-dependent manner (Rozalen et al, 2025). Using a dual fluorescent reporter for metastatic burden and transcription from the *Havcr2* promoter, Rozalén and collaborators next demonstrated that while the majority of 4T07 cells reaching metastatic sites in immunocompetent animals die short after intracardiac inoculation, the TIM-3^+^ population persists and effectively drives the transition from micro- to macrometastasis, a dissemination dynamic that was confirmed in other immunocompetent mouse TNBC models, but not in immunodeficient hosts, further confirming an immunological selection process. Notably, the immunological microenvironment of TIM-3^+^ 4T07 micrometastases was selectively enriched in IL-17^+^ γδ T cells (which have previously been shown to promote metastasis in TNBC models) (Coffelt et al, 2015), and CD8^+^ cytotoxic T lymphocytes (CTLs) expressing the co-inhibitory receptor programmed cell death 1 (PDCD1, best known as PD-1), coupled with the depletion of CD8^+^ CTLs expressing activation markers including granzyme B (GZMB) and CD69 (Rozalen et al, 2025).

Next, Rozalen et al (2025) performed in vivo functional assays based on monoclonal antibodies with blocking or depleting activity aimed at different immune cell populations. While depleting natural killer (NK) cells, CD8^+^ CTLs, CD4^+^ T cells, B cells or neutrophils had no effect on the ability of TIM-3-expressing 4T07 TNBC cells to form liver micrometastases upon intracardiac delivery to BALB/c mice, blocking γδ TCR signaling reduced it as effectively as TIM-3 depletion in malignant cells. Such an effect, however, was less pronounced in other organs and not associated with an overall survival advantage, suggesting a selective role of IL-17^+^ γδ T cells in the establishment of hepatic (but not extrahepatic) metastases. Of note, IL-17^+^ γδ T cells appeared to accumulate in the micrometastatic environment established by 4T07 TNBC cells in an IL-1β-dependent manner, and IL-1β depletion from the malignant compartment decreased metastatic load in the liver (Rozalen et al, 2025). To investigate the pathophysiological implications of their findings, Rozalén and colleagues interrogated the impact of TIM-3 expression by malignant cells on breast cancer progression in a tissue microarray of 257 primary samples. This revealed that elevated TIM-3 expression by breast cancer cells is associated with shortened disease-specific and overall survival, as well as with an increased probability of relapse that remained significant on multivariate survival analysis. The link between increased TIM-3 expression and poor disease outcome appears to be causal, as TIM-3 blockade effectively prevented metastatic dissemination in both the intracardiac model with 4T07 cells, as well as in the spontaneous 4T1 model (in a neoadjuvant treatment schedule) (Rozalen et al, 2025).

**Figure 1:**
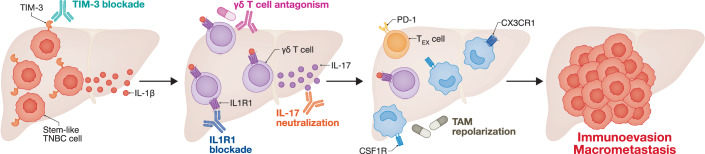
TIM-3^+^ TNBC cells establish hepatic metastases via IL-17^+^ γδ T cells. Compared to their TIM-3^-^ counterparts, TIM-3^+^ triple-negative breast cancer (TNBC) cells exhibit stemness traits including signs of the epithelial-to-mesenchymal transition (EMT) and WNT/β-catenin signaling, which endow them with superior immunoevasive properties. Accordingly, TIM-3^+^ TNBC cells preferentially survive immunological eradication upon reaching metastatic sites, resulting in the efficient establishment of micrometastatic disease. At least in the liver, such TIM-3^+^ TNBC micrometastases produce IL-1β, resulting in the recruitment of a population of IL-17^+^ γδ T cells that aggravate local immunosuppression, *de facto* driving metastatic outgrowth. Whether such an immunosuppressive effect relies on CX3CR1^+^ tumor-associated macrophages (TAMs), as in other breast cancer subtypes, however, remains to be formally investigated. Despite such an unknown, this immunological mechanism offers a number of clinically viable targets for intervention, including (but not limited to): TIM-3 blockers, IL-1β or IL-17 signaling inhibitors, and perhaps TAM-repolarizing agents. CSF1R colony-stimulating factor 1 receptor, IL1R1 interleukin 1 receptor type 1, T_EX_ exhausted T.

In summary, Rozalen et al (2025) delineated a new mechanism through which TIM-3^+^ TNBC cells, which exhibits stem cell-like traits and hence increased resistance to immunological eradication, establish a micrometastatic environment enriched in IL-17^+^ γδ T cells that support metastatic outgrowth and overt immune evasion (Fig. 1). Recently, IL-17^+^ γδ T cells have been shown to promote resistance to CDK4/6 inhibitors in hormone receptor (HR)^+^ breast cancer, at least in part by favoring the repolarization of tumor-associated macrophages (TAMs) toward an immunosuppressive CX3CR1^+^ phenotype (Buque et al, 2020; Petroni et al, 2025). Rozalén and colleagues (2025) also detected a selective enrichment of TAMs in the micrometastatic environment of TIM-3^+^ TNBCs. Whether these TAMs expressed CX3CR1 and mechanistically contributed to metastatic outgrowth, however, was not specifically investigated. Despite this and other unknowns, targeting TIM-3, IL-1β receptors, IL-17^+^ γδ T cells, IL-17 and their downstream immunological effectors (perhaps including CX3CR1^+^ TAMs) (Paul et al, 2024) represent promising strategies to limit metastatic dissemination and resistance to therapy in multiple breast cancer subtypes. Clinical studies formally addressing these options are urgently awaited.
